# Patient Satisfaction and Treatments Offered to Swedish Patients with Suspected Pediatric Acute-Onset Neuropsychiatric Syndrome and Pediatric Autoimmune Neuropsychiatric Disorders Associated with Streptococcal Infections

**DOI:** 10.1089/cap.2018.0141

**Published:** 2019-10-07

**Authors:** Eva Hesselmark, Susanne Bejerot

**Affiliations:** ^1^ Department of Clinical Neuroscience, Center for Psychiatry Research, Karolinska Institutet, Stockholm, Sweden.; ^2^ Stockholm Health Care Services, Stockholm County Council, Stockholm, Sweden.; ^3^ School of Medical Sciences, Örebro University, Örebro, Sweden.; ^4^ Faculty of Medicine and Health, University Health Care Research Center, Örebro University, Örebro, Sweden.

**Keywords:** pediatric acute-onset neuropsychiatric syndrome, pediatric autoimmune neuropsychiatric disorders associated with streptococcal infections, treatment outcome, obsessive-compulsive disorder, patient satisfaction

## Abstract

***Objectives:*** Pediatric acute-onset neuropsychiatric syndrome (PANS) and pediatric autoimmune neuropsychiatric disorders associated with streptococcal infections (PANDAS) are subtypes of Obsessive-Compulsive Disorder (OCD) with suggested autoimmune etiology. Immunomodulatory treatments have been introduced as treatment options. A recent systematic review concluded that the evidence for all treatment options for PANS and PANDAS is inconclusive. However, case reports and clinical experience suggest that antibiotics and immunomodulatory treatment may be helpful. Treatment may also affect the patients' satisfaction with health care services offered. This study aims to describe the treatments given to a cohort of Swedish patients with suspected PANS and PANDAS, the patient rated treatment effects, and to establish if any specific treatment predicts higher patient satisfaction.

***Methods:*** Fifty-three patients (m = 33, f = 20, median age = 14, age range = 4–36) with suspected PANS or PANDAS were enrolled and assessed for PANS and PANDAS caseness, treatments given, treatment effects, global improvement, and patient satisfaction. Cases with confirmed and suspected PANS or PANDAS were compared regarding the frequency of treatments given and treatment effect. A linear regression model was used to see if treatments given or global improvement predicted patient satisfaction.

***Results:*** Twenty-four participants fulfilled criteria for PANS or PANDAS and 29 did not. The most common treatments given were antibiotics (88%), nonsteroidal anti-inflammatory drugs (67%), cognitive behavioral therapy (53%), and selective serotonin reuptake inhibitors (42%). There were no major differences between confirmed and suspected cases regarding what treatments they had received or their effect. Patient satisfaction was predicted by overall clinical improvement at the time of assessment. Antibiotics and intravenous immunoglobulin (IVIG) were rated as the most successful treatments by participants and were associated with higher patient satisfaction.

***Conclusions:*** It was more common that patients had received antibiotics than common psychiatric treatments for their psychiatric symptoms. Antibiotics and IVIG were experienced as effective treatments by the patients. Patient satisfaction was on average moderately low, and higher patient satisfaction was associated with global clinical improvement.

## Introduction

Obsessive-compulsive disorder (OCD) is a neuropsychiatric disorder characterized by obsessions and compulsions (American Psychiatric Association 2013). The exact etiology of OCD is currently unknown, but it is probable that genetic, as well as environmental, risk factors play a role (Mataix-Cols et al. 2013). Recent literature has proposed a possible link between OCD and autoimmune disorders (Mataix-Cols et al. 2018) and OCD and streptococcal infection (Perez-Vigil et al. 2016; Orlovska et al. 2017). OCD can be treated with cognitive behavioral therapy (CBT), including exposure with response prevention and with selective serotonin reuptake inhibitors (SSRIs) (National Institute for Health and Clinical Excellence 2005). These treatments are highly effective, although not all patients experience remission (Skapinakis et al. 2016).

Pediatric acute-onset neuropsychiatric syndrome (PANS) (Swedo et al. 2012) and pediatric autoimmune neuropsychiatric disorders associated with streptococcal infections (PANDAS) (Swedo et al. 1998) are two subtypes of OCD with suggested autoimmune etiology. PANDAS is proposed to have a pathophysiology similar to that of Sydenham's chorea; a streptococcal infection causes an autoimmune disorder through the production of antibodies that cross-react to both streptococcus and brain tissue (Garvey et al. 1998; Swedo et al. 1998). PANS does not have a specific proposed pathophysiology, but is nevertheless thought to have an autoimmune component (Swedo et al. 2012).

Because PANS and PANDAS may be autoimmune disorders, a multitude of treatment options related to this pathophysiology have been introduced clinically (Cooperstock et al. 2017; Frankovich et al. 2017). Antibiotics have been used to treat streptococcus to ameliorate psychiatric symptoms (Cooperstock et al. 2017). Immunomodulatory drugs such as corticosteroids, intravenous immunoglobulin (IVIG), rituximab, mycophenolate mofetil, and nonsteroidal anti-inflammatory drugs (NSAIDs) have also been tried (Frankovich et al. 2017). A recent systematic review on PANS and PANDAS treatment concluded that the evidence for all treatment options is inconclusive (Sigra et al. 2018). However, there are many case reports and much clinical experience that suggest that antibiotics and immunomodulatory treatment may be helpful for patients with PANS and PANDAS (Sigra et al. 2018).

Although the immunological approach to PANS and PANDAS currently lacks strong evidence, immunological treatments and antibiotics may be beneficial and they are also often requested by patients. However, as the diagnoses are debated, and often unfamiliar to the clinicians, these treatments are not regularly prescribed. Patients may need to contact several health care providers to be identified as PANS or PANDAS cases and obtain, for example, antibiotics or immunomodulatory treatments. This treatment delay may result in low confidence for the health care system and poor patient satisfaction.

The aim of this study is to describe the treatments given to a cohort of Swedish patients with suspected PANS and PANDAS, and their effects. A secondary aim is to establish if specific treatments predict higher patient satisfaction.

## Methods

### Study design

This is a *post hoc* analysis of data collected within the study “PANS—A detailed study of the patients, their symptoms, biomarkers, and treatment offered in a Scandinavian cohort,” which was registered before enrollment of participants; Clinicaltrials.gov; NCT02190292.

The aim of the recruitment procedure was to be able to compare patients with PANS to psychiatric patients who did not fulfill criteria for PANS. Because one of the aims of the larger study was to evaluate the diagnostic value of a blood test aimed at diagnosing PANS or PANDAS, the recruitment and inclusion followed Standards for Reporting of Diagnostic Accuracy Studies guidelines for studies of diagnostic accuracy (Bossuyt et al. 2015). These guidelines require that the diagnostic test can differentiate between true cases and patients who are likely to be assessed for the disorder in the clinic. The blood test evaluated is called the Cunningham Panel (Moleculera 2016), and the methods and result of this study are described in a previous article (Hesselmark and Bejerot 2017). The Cunningham Panel comprises five analytes measured in serum: calcium/calmodulin dependent kinase II activation, Dopamine receptor D1 and D2 antibodies, β-tubulin antibodies, and lysoganglioside antibodies (Moleculera 2016) and was developed by Moleculera Labs, Oklahoma City, OK.

At the time of inclusion, the name used for Cunningham Panel in Sweden was “PANDAS-panelen” or the “PANDAS panel.” The panel is costly, and it could only be ordered from one specific laboratory (Wieslab). The panel also had to be ordered by a medical doctor (self-referrals were not accepted by Wieslab). Thus, it was concluded that patients who had taken the panel had been suspected of having PANS or PANDAS by the physician who ordered the test. By inviting all patients who had taken the test (regardless of their test results) and then assessing them for PANS and PANDAS criteria (while being blind to their test results), two groups were formed: one group who fulfilled PANS or PANDAS criteria (Interview Confirmed PANS), and one group who was clinically similar, but who did not fulfill criteria (Suspected PANS).

### Participants and recruitment

**FIG. 1.**
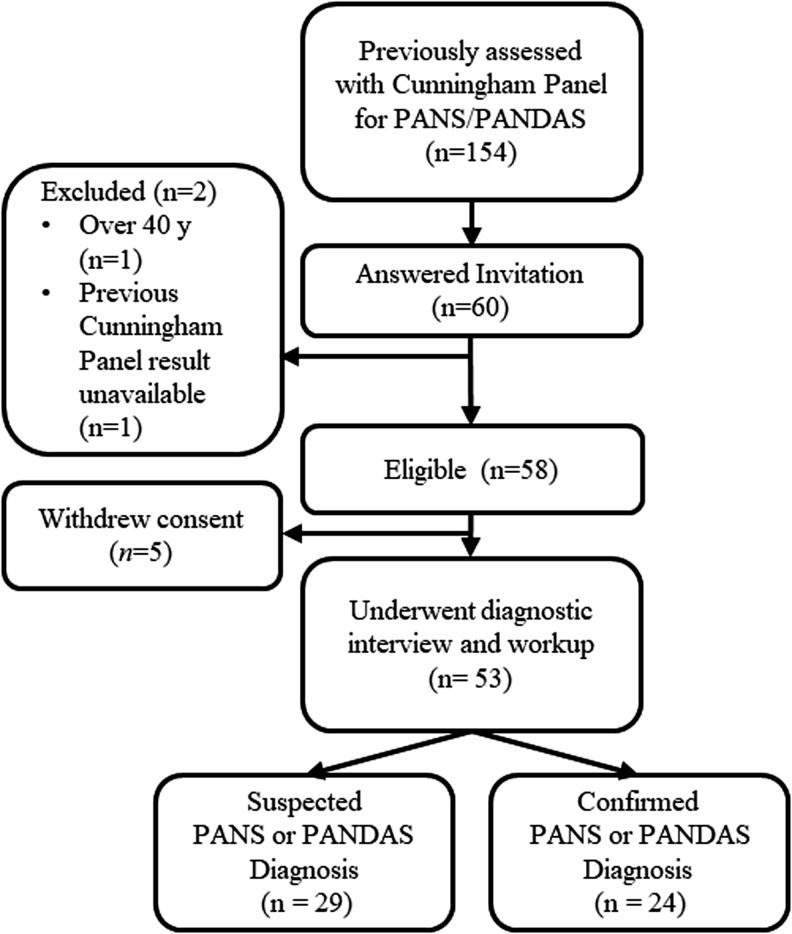
Flow chart of participant inclusion and classification.

All patients (*n* = 154) who had taken the Cunningham Panel in Sweden since the first available sample (April 2013) to study start (June 2014) were invited to participate. This inclusion criterion allowed us to recruit a sample of patients who had been suspected of having PANS or PANDAS by their treating doctor. Exclusion criteria were age over 40 years or inability to complete the assessment in Swedish. Fifty-three patients (m = 33, f = 20, median age = 14 years, age range = 4–36 years) agreed to participate and were enrolled. The 101 nonparticipants (who were invited to the study, but declined to participate) had similar age, gender, and Cunningham Panel results. See Figure 1 for enrollment flowchart. Details on the materials and methods are described in a previous study (Hesselmark and Bejerot 2017).

### Procedure

Each participant was interviewed on one occasion. The aim of the interview was to assess psychiatric symptoms and general function, PANS and PANDAS caseness, treatments received, treatment effects, and patient satisfaction. Parents were invited to assist in the interview where appropriate and to provide data on developmental and treatment history. The ages of participants in this study vary between 4 and 36 years, and each interview was made with individual consideration of each participant's ability to complete the assessment. Adult patients with intelligence within the normal range and ability to partake in the interview rated most of their own symptoms and treatment responses. Small children and patients with severe psychiatric symptoms were less able to rate their own symptoms, and we therefore relied more on the parents' reports of symptoms and treatment outcomes. In this article, we will refer to all self- or parent rated measures as “patient rated.” Each interview took between 3 and 5 hours. All interviews were performed by the authors of this study: a senior psychiatrist (S.B.) and a PhD candidate trained in psychology (E.H.). The interviews occurred in 2015 and 2016 at an outpatient setting local to the participant or in the participants' homes.

### Measures

#### Psychiatric assessment and classification of PANS and PANDAS

Each interview included structured psychiatric tools such as The Mini International Neuropsychiatric Interview (M.I.N.I.) (Sheehan et al. 1998) for adults and M.I.N.I.—KID (Sheehan et al. 2010) for children and the Yale Brown Obsessive Compulsive Scale (Y-BOCS) (Goodman et al. 1989) for adults and Children's Yale Brown Obsessive Compulsive Scale (CY-BOCS) (Scahill et al. 1997) for children. We also included a global measure of function, the Clinical Global Impressions-Severity (CGI-S) (Guy 1976), which was rated by the clinician at the end of each interview. After each interview the two assessors used diagnostic criteria for PANS (Swedo et al. 2012) and PANDAS (Swedo et al. 1998) and used all available information to classify each patient as fulfilling criteria for PANS or PANDAS, both or neither. “Acute-onset”—a prerequisite for these diagnoses—was defined as having <72 hours between onset and fulminant psychiatric symptoms. Tourette disorder was defined as an exclusion criterion for PANS in the original diagnostic criteria (Swedo et al. 2012), but was not used as such in this study. Patients who met the criteria of PANS and PANDAS or both were classified as Confirmed cases in this study. Patients who did not meet the criteria for either PANS or PANDAS were classified as Suspected cases.

#### Assessment of treatment and patient rated treatment effect

All participants were asked whether or not they had received specific treatments for their psychiatric disorders, PANS or PANDAS. Treatments included a wide range of psychiatric and immunomodulatory drugs, antibiotics, and nonpharmacological treatments such as psychotherapy and dietary change. Participants were asked to rate the effect on a four-point Likert scale of each received treatment (worse, no effect, small improvement, or much improved). For the analysis the responses “no effect” and “small improvement” were coded into one category named “small or no effect,” which resulted in three possible ratings of effect: “worse,” “small or no effect,” and “much improved.” Some participants reported that they had received a specific treatment, but did not rate the effect. These responses were coded as “unknown effect.”

We also included a general self-rated measure of global improvement, by letting the patient rate themselves with the Clinical Global Impressions-Improvement (CGI-I) (Guy 1976) scale. The CGI-I is a widely used one item scale designed to measure change in symptoms. The scale ranges from 1 = “very much improved” to 7 = “very much worse,” with 4 being a neutral score of “no change.” The scale was developed as a clinician rated scale, but in this study, it is rated by the participant. At the time of our assessment, the patients were asked how much they had improved since the first Cunningham Panel test, taken before June 2014.

#### Assessment of patient satisfaction

The Client satisfaction questionnaire (CSQ) was used to measure global patient satisfaction (Larsen et al. 1979). The CSQ comprises eight items and is a commonly used instrument to measure patients' satisfaction within clinical care. The items are phrased as questions such as “How would you rate the quality of the service you received?” and each item is rated on an individual scale from 1 to 4 (e.g., poor = 1; fair = 2; good = 3; excellent = 4). The items cover quality of service, if the patient got the service they wanted, if the service met the patient's needs, if the patient would recommend the service to others, if the patient is satisfied with the services and with the amount of services, if the service has helped, and if the patient would seek help in the same place again. CSQ ranges from 8 to 32 points, and higher scores indicate higher satisfaction. Scores can be grouped into low (8–20), medium (21–26), or high (27–32) patient satisfaction (Larsen et al. 1979). In this study the instrument was used at the time of our assessment. Participants were instructed to globally evaluate the received clinical care, since the onset of the PANS and PANDAS related symptoms (i.e., OCD, tics, eating disorder, and so on).

### Statistics

To analyze if some treatments were more commonly prescribed for participants with Confirmed PANS or PANDAS than Suspected PANS or PANDAS, we compared the two groups using χ^2^-tests. We also used χ^2^-tests to compare the relative frequency of participants who rated their response to be “much improved” compared with “worse” or “no or little effect.” Comparisons between groups regarding self-rated treatment effect were only made if at least 25% of participants had received the treatment.

To determine if patient satisfaction was related to the specific treatments given or to general outcome, we first examined if there was a correlation between patient-rated CGI-I and CSQ. We then made regression models to determine if higher CSQ was predicted by treatment with antibiotics, IVIG, NSAIDs, CBT, or SSRIs, respectively. In a *post hoc* analysis patient-rated CGI-I was added to the regression model of any significant results, to see if the relationship between specific treatments and CSQ was driven mainly by clinical improvement.

All data were analyzed using SPSS version 23. The Bonferroni method was used to adjust for multiple comparisons within each analysis. Demographic data are presented as medians or proportions.

### Ethical considerations

All study participants and/or legal guardians granted informed consent. Our protocol was approved by the Regional Ethics Review Board of Stockholm (2014/551-31/2; 2014/1711-32; 2015/964-31; 2016/2121-32).

## Results

### Inclusion and demographics

A total of 154 patients had taken the Cunningham Panel before study enrollment and were thus invited to participate in the study. Sixty patients responded to the invitation, 53 of whom chose to participate and underwent the diagnostic interview for PANS and PANDAS. See Figure 1 for inclusion flowchart. Median age at disorder onset was 7.9 years (range 1–20), and median age at time of our assessment was 14 years (range 4–36). Participants were markedly ill at the time of assessment with a median CGI-S score of 5 (range 2–7). The median CSQ score was 17 (range = 8–32), which indicates mild dissatisfaction with the health care services received. For detailed demographic data, see Table 1.

**Table 1.** Demographic Information on Included Patients (*n* = 53)

*Description*	*Value*	*Missing* n
Age at assessment, median (range)	14 (4–36)	0
Age at onset, median (range)	7.9 (1–20)	1
Female, *n* (%)	20 (38)	0
Confirmed PANS or PANDAS, *n* (%)	24 (45)	0
Diagnoses according to M.I.N.I., median (range)	6 (1–20)	0
Y-BOCS or CY-BOCS score, median (range)	13 (0–39)	11
CGI-S at time of assessment, median (range)	5 (2–7)	3
CGI-I since first Cunningham Panel result, median (range)	3.0 (1–6)	3
CSQ total score, median (range)	17 (8–32)	8

CGI-I, Clinical Global Impressions-Improvement; CGI-S, Clinical Global Impressions-Severity; CSQ, Client satisfaction questionnaire; CY-BOCS, Children's Yale Brown Obsessive Compulsive Scale; M.I.N.I., Mini International Neuropsychiatric Interview; PANDAS, pediatric autoimmune neuropsychiatric disorders associated with streptococcal infections; PANS, pediatric acute-onset neuropsychiatric syndrome; Y-BOCS, Yale Brown Obsessive Compulsive Scale.

### Treatment given and patient rated treatment effect

**FIG. 2.**
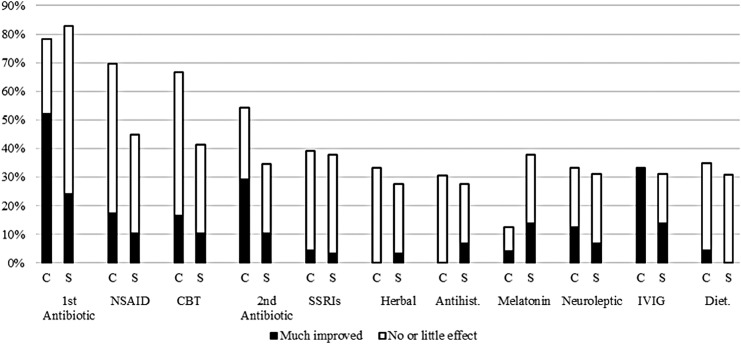
Frequencies of patients who had received treatments for their psychiatric symptoms and the treatment effect. C, confirmed PANS or PANDAS; CBT, cognitive behavioral therapy; Diet., dietary change; IVIG, intravenous immunoglobulin; NSAID, nonsteroidal anti-inflammatory drug; PANDAS, pediatric autoimmune neuropsychiatric disorders associated with streptococcal infections; PANS, pediatric acute-onset neuropsychiatric syndrome; S, suspected PANS or PANDAS; SSRIs, selective serotonin reuptake inhibitors.

All participants had received some kind of treatment for their psychiatric symptoms. There were no major differences between the Confirmed PANS group and the Suspected PANS group in regard to the received treatments or patient rated treatment effect. In the Confirmed PANS group 87% of the participants had received at least one type of antibiotics, and 58% had received at least two types. Furthermore, 78% had been treated with NSAIDs, 33% had received IVIG, 67% had received CBT, and 48% had received SSRIs. The frequencies of treatments in the Suspected PANS group were similar; 90% received at least one antibiotic treatment, and 31% received IVIG. The treatments with the best patient rated effects were IVIG (12 out of 17 treated participants [71%] report to be “much better”) and antibiotics (19 out of 46 treated participants [41%] report to be “much better”). For detailed data, please see Table 2. When controlling for multiple testing, there were no significant differences between the patient rated treatment effects in the two groups. It was uncommon for participants to indicate that treatments made them “worse,” but six patients indicated that they were “worse” when treated with SSRIs, six when treated with oral neuroleptics, and six when treated with central stimulants. See Figure 2 and Supplementary Table S1 for details.

**Table 2.** Total Number of Patients Who Received Each Treatment in the Two Groups, *N* (%)

	*Confirmed PANS/PANDAS (*n* = 24)*		*Suspected PANS/PANDAS (*n* = 29)*		*Total sample (*n* = 53)*	
	N *(%)*	^a^	N *(%)*	^a^	N *(%)*	^a^
Antibiotics (first)	20 (87)	1	26 (90)	0	46 (88)	1
NSAIDs	18 (78)	1	17 (59)	0	35 (67)	1
CBT	16 (67)	0	13 (45)	0	28 (53)	0
Antibiotics (second)	14 (58)	0	10 (34)	0	23 (43)	0
SSRIs	10 (48)	1	14 (48)	0	22 (42)	1
Herbal medicine	10 (48)	3	12 (41)	0	21 (42)	3
Antihistamines	9 (39)	1	11 (38)	0	20 (38)	1
Melatonin	4 (17)	0	15 (52)	0	19 (36)	0
Neuroleptics oral	10 (42)	0	10 (34)	0	18 (34)	0
IVIG	8 (33)	0	9 (31)	0	17 (32)	0
Dietary change	8 (33)	1	8 (31)	3	15 (31)	4
Corticosteroids oral	5 (22)	1	5 (17)	0	10 (19)	1
Central stimulants	5 (21)	0	8 (28)	0	10 (19)	0
Tonsillectomy	6 (29)	3	3 (11)	1	9 (18)	4
Sleep medication	3 (13)	0	4 (14)	1	7 (13)	1
Adenoidectomy	2 (9)	2	3 (10)	0	5 (10)	2
Benzodiazepines	1 (4)	1	4 (14)	1	5 (10)	2
Antibiotics (third)	3 (13)	0	2 (7)	0	5 (9)	0
Mood stabilizers	2 (8)	0	3 (10)	0	5 (9)	0
N-acetylcysteine	1 (4)	0	3 (11)	1	4 (8)	1
Hormonal treatment	1 (4)	1	2 (7)	0	3 (6)	1
Anxiety management	2 (8)	0	0 (0)	0	2 (4)	0
Corticosteroids IV/IM	1 (4)	0	1 (3)	0	2 (4)	0
Rituximab	1 (4)	0	1 (3)	0	2 (4)	0
SNRIs	0 (0)	3	1 (3)	0	1 (2)	3
Lithium	0 (0)	0	1 (3)	0	1 (2)	0
Neuroleptics IV/IM	0 (0)	0	1 (3)	0	1 (2)	0

^a^ Missing data.

CBT, cognitive behavioral therapy; IVIG, intravenous immunoglobulin; IV/IM, intravenous/intramuscular; NSAID, nonsteroidal anti-inflammatory drug; PANDAS, pediatric autoimmune neuropsychiatric disorders associated with streptococcal infections; PANS, pediatric acute-onset neuropsychiatric syndrome; SNRIs, serotonin–norepinephrine reuptake inhibitors; SSRIs, selective serotonin reuptake inhibitors.

### Relationship between treatments given, treatment effect, and patient satisfaction

**FIG. 3.**
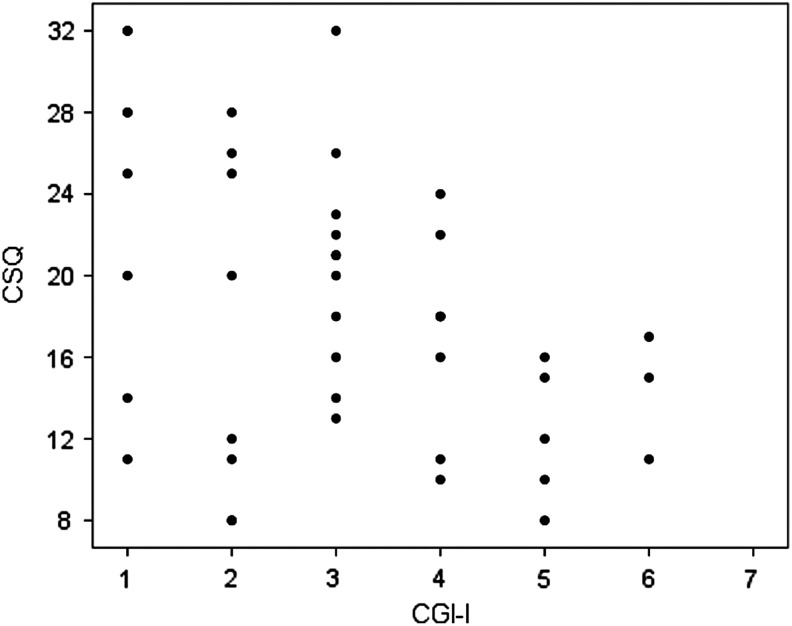
Plot of correlation between patient satisfaction measured with the CSQ and clinical improvement measured with a patient or parent rated CGI-I. CSQ is scored 8–32, and a high score indicates high satisfaction. CGI-I is scored 1–7, where 1 is “very much improved,” 4 is “no change,” and 7 is “very much worse.” CGI-I, Clinical Global Impressions-Improvement; CSQ, Client satisfaction questionnaire.

Patient rated global improvement measured with CGI-I significantly predicted patient satisfaction measured with the CSQ, with high satisfaction being associated with larger improvement [*B* = −0.426, *t*(41) = 2.976, *p* = 0.005] (Fig. 3). CSQ scores were not predicted by treatment with SSRIs [*B* = 0.142, *t*(44) = 0.94, *p* = ns], NSAIDs [*B* = 0.09, *t*(44) = 0.576, *p* = ns], or CBT [*B* < 0.01, *t*(44) = 0.034, *p* = ns]. CSQ scores were however associated with IVIG treatment, with patients treated with IVIG indicating higher patient satisfaction [*B* = 0.363, *t*(44) = 2.555, *p* = 0.01] and similarly associated with antibiotics [*B* = 0.30, *t*(44) = 2.097, *p* = 0.04]. When controlling for global improvement measured with CGI-I, neither IVIG [*B* = 0.263, *t*(41) = 1.806, *p* = 0.08] nor antibiotics [*B* = 0.208, *t*(18) = 1.349, *p* = 0.19] predicted patient satisfaction.

## Discussion

We asked 53 participants who had been suspected of having PANS or PANDAS about their symptoms, medical history, treatments, treatment effect, and patient satisfaction. Twenty-four participants fulfilled criteria for PANS or PANDAS and 29 did not. There were no major differences between confirmed and suspected cases regarding what treatments they had received or their effect. Participants were mildly dissatisfied with the health care services they had received, and patient satisfaction was predicted by overall clinical improvement at the time of assessment. Treatment with IVIG predicted higher patient satisfaction.

### Treatments received and patient rated treatment effect

Patients in both the Confirmed and the Suspected PANS groups had received similar treatments. Notably, only 53% of the participants had received CBT. CBT is recommended for treatment of OCD, as well as for anxiety and depression, all common disorders among our participants. In contrast, only 7 of the 29 participants (24%) who had received CBT reported it to have a satisfactory effect. Similarly, only 46% of the patients had received an SSRI, which is equally surprising since SSRIs are considered first line pharmacological treatment for OCD, anxiety, and depression. SSRIs were also reported to have a nonsatisfactory effect. Only 2 of the 24 (8%) participants who had received SSRIs reported them to be beneficial, and 6 of 24 (25%) recipients reported that SSRIs aggravated their symptoms. These unexpected findings are difficult to interpret. On the one hand, it could be argued that suspicion of PANS or PANDAS may lead to underuse of evidence based treatment for psychiatric disorders such as OCD, depression, and anxiety. On the other hand, our participants reported poor effect from these treatments, and previous reports have also stated that SSRIs may deteriorate PANDAS (in 2012 it was recommended to “start low and go slow” when using SSRIs to treat PANS and PANDAS because of the risk of side effects) (Swedo et al. 2012). However, since SSRIs and CBT are evidence-based treatments for OCD, depression, and anxiety, they may be recommended first line treatments before introducing immunomodulatory drugs (Gilbert et al. 2018).

The eight Confirmed PANS or PANDAS participants treated with IVIG reported to be much improved compared with four out of nine participants in the Suspected PANS/PANDAS group. However, this difference between groups was not statistically significant when the analysis was controlled for multiple testing. In our recent review of treatment studies for PANS and PANDAS, we reported that the evidence for IVIG in PANS and PANDAS is inconclusive. Two double-blind randomized controlled trials of IVIG for PANDAS have hitherto been published. The results are conflicting; one indicates a good effect (Perlmutter et al. 1999), whereas the other one failed to prove IVIG superiority over placebo (Williams et al. 2016). However, in a large survey study 49% of patients with PANS treated with IVIG reported IVIG to be “very effective” (Calaprice et al. 2018).

### Relationship between treatments, treatment effect, and patient satisfaction

In our sample the measure of global improvement predicted patient satisfaction to a large degree (*r* = 0.43), which is expected and in line with the original article on CSQ (Pearson correlation *r* = 0.53) (Larsen et al. 1979). Moreover, the level of patient satisfaction among our participants was similar to patients with other psychiatric diagnoses in Sweden (Lenander 2018). In our sample, treatment with IVIG and antibiotics predicted higher patient satisfaction scores, although this effect was driven by global improvement. As IVIG lacks indication for treatment of psychiatric disorders, in addition to being invasive and costly, we assume that physicians are reluctant to prescribe IVIG on psychiatric indication and only do so after a thorough assessment. Moreover, IVIG treatment requires day-hospital care and a multidisciplinary team as the treatment is usually administered by a neurologist or immunologist. This thorough assessment and multidisciplinary approach, in combination with the intravenous administration of the treatment, may give patients a sense of being listened to and being taken seriously. The IVIG treatment may be experienced as a sign of the health care professionals' willingness to do everything in their power to help. This experience may be the reason for the higher levels of patient satisfaction seen among IVIG receivers.

### Limitations

This study includes a number of limitations regarding the measures used. This is a retrospective study based on parental and patient reports; therefore, the measure of treatment effect is inexact and possibly biased. The CSQ was developed to evaluate a specific treatment given by a specific health care provider and in this study it was used as a general measure of satisfaction of all previous health care interventions. However, this usage is supported by the expected correlation between global improvement and the CSQ. The CGI-I used was an unvalidated patient- or parent reported version, while the scale was developed to be rated by a clinician (Guy 1976); therefore, the CGI-I scores in this study may be unreliable. However, the symptomatology of PANS and PANDAS is complex; thus, parents may have a better appreciation of treatment effects than a clinician who only sees the patient occasionally.

Furthermore, we did not collect data on the timing of treatments and therefore do not know if the patients received several treatments simultaneously. It is highly probable that many participants received several simultaneous treatments due to the nature of the disorders with multiple symptoms. However, our results are similar to a larger survey study (*n* = 698) of patient rated treatment response in PANS and PANDAS (Calaprice et al. 2018).

The study sample is small, with only 24 and 29 participants in the respective study groups and only a proportion of those having received each of the studied treatments. This resulted in a lack of statistical power for many of the comparative analyses, and thus, the nondifference between groups regarding the treatment effect may be due to the small sample size.

The study sample also has some characteristics that are of importance to the interpretation of our results. The inclusion criterion for the study was to have taken the Cunningham Panel before June 2014. We believe that patients with PANDAS or PANDAS-like symptoms that respond to evidence based psychiatric treatment, such as SSRIs and CBT, are unlikely to have been offered the Cunningham Panel. Therefore, our sample possibly includes severe cases with poor treatment outcomes and, thus, may not be representative regarding patient satisfaction or treatment outcome. This may also lead to an overestimation of the risk of undertreatment with CBT and SSRIs reported here. We do not know if our sample is representative of the 154 patients that had been tested with the Cunningham Panel, but we know that the nonparticipants (*n* = 101) had similar age, gender, and Cunningham Panel results to the participants (*n* = 53), which indicates representativeness (Hesselmark and Bejerot 2017).

## Conclusion

The patients in our study had been offered a large number of treatments over the years, which reflect a desperation for any amelioration of severe treatment resistant symptoms. Patients with PANS and PANDAS may be undertreated with established psychiatric interventions such as SSRIs and CBT, although these treatments are not always experienced as successful. Patient satisfaction was on average moderately low, and a higher patient satisfaction was associated with a better global outcome.

## Clinical Significance

PANS and PANDAS are severe and complex psychiatric disorders. When treating patients with suspected or confirmed PANS or PANDAS, it is important to have knowledge about both psychiatric and immunomodulatory treatments. Patients with PANS and PANDAS are at risk of experiencing poor treatment outcomes and low satisfaction with the offered health care service. Therefore, it is important that clinicians carefully monitor and follow treatments with regards to outcomes and side effects. Since there is no strong evidence for any specific treatment in PANS and PANDAS, it is a challenge for the psychiatrist to satisfy the patient and their families with available treatment options. We suggest that it may be important for patient satisfaction to carefully offer information and rationale, both when offering and not offering specific treatments. Finally, there is clearly need for more high quality studies regarding the treatment of PANS and PANDAS.

## Supplementary Material

Supplemental data
