# Supplementary material for: Patient Satisfaction and Treatments Offered to Swedish Patients with Suspected Pediatric Acute-Onset Neuropsychiatric Syndrome and Pediatric Autoimmune Neuropsychiatric Disorders Associated with Streptococcal Infections
Source: J Child Adolesc Psychopharmacol. 2019 Oct 7;29(8):634–41. doi: 10.1089/cap.2018.0141 (PMC6786336; doi:10.1089/cap.2018.0141)
Supplement: Supplemental data [file Supp_Table1.pdf]

Supplementary Data

Supplementary Table S1. Full Data on Treatment and Treatment Effect

Treatment	*Confirmed PANS or PANDAS* (n=24)								*Suspected PANS or PANDAS* (n=29)							
	No treatment	*Worse*	*No effect*	*Little better*	*Much better*	*Unknown effect*	*Total*	*Missing data*	*No treatment*	*Worse*	*No effect*	*Little Better*	*Much better*	*Unknown effect*	*Total*	*Missing data*
Antibiotics (first)	3	0	3	3	12	2	23	1	3	1	8	8	7	2	29	0
NSAIDs	5	0	5	7	4	2	23	1	12	0	3	7	3	4	29	0
CBT	8	1	6	5	4	0	24	0	16	0	4	5	3	1	29	0
Herbal medicine	11	1	5	1	0	3	21	3	17	0	4	3	1	4	29	0
SSRIs	13	2	5	1	1	1	23	1	15	4	2	4	1	3	29	0
Antibiotics (second)	10	1	2	3	7	1	24	0	19	1	1	5	3	0	29	0
Antihistamines	14	0	2	5	0	2	23	1	18	1	3	2	2	3	29	0
Dietary change	15	1	3	3	1	0	23	1	18	0	4	4	0	0	26	3
Neuroleptics Oral	14	2	2	1	3	2	24	0	19	4	1	2	2	1	29	0
Melatonin	20	0	0	2	1	1	24	0	14	0	6	1	4	4	29	0
IVIG	16	0	0	0	8	0	24	0	20	0	1	4	4	0	29	0
Central stimulants	19	3	1	0	0	1	24	0	21	3	2	1	2	0	29	0
Tonsillectomy	15	0	3	2	1	0	21	3	25	0	2	1	0	0	28	1
Corticosteroids oral	18	0	2	1	1	1	23	1	24	0	1	0	2	2	29	0
Sleep medication	21	0	0	1	0	2	24	0	24	0	1	0	0	3	28	1
Adenoidectomy	20	0	1	0	1	0	22	2	26	0	2	1	0	0	29	0
Benzodiazepines	22	0	0	0	0	1	23	1	24	1	2	1	0	0	28	1
Antibiotics (third)	21	0	0	1	1	1	24	0	27	0	1	0	0	1	29	0
Mood stabilizers	22	0	0	0	1	1	24	0	26	0	1	1	0	1	29	0
N-acetylcysteine	23	0	1	0	0	0	24	0	25	0	1	1	0	1	28	1
Hormonal treatment	22	0	0	0	0	1	23	1	27	0	1	0	0	1	29	0
SNRIs	21	0	0	0	0	0	21	3	28	0	0	1	0	0	29	0
Anxiety management	22	0	1	1	0	0	24	0	29	0	0	0	0	0	29	0
Corticosteroids IV/IM	23	0	0	1	0	0	24	0	28	1	0	0	0	0	29	0
Rituximab	23	0	0	1	0	0	24	0	28	1	0	0	0	0	29	0
Lithium	24	0	0	0	0	0	24	0	28	0	0	0	0	1	29	0
Neuroleptics IV/IM	24	0	0	0	0	0	24	0	28	1	0	0	0	0	29	0

CBT, cognitive behavioral therapy; IVIG, intravenous immunoglobulin; IV/IM, intravenous/intramuscular; NSAID, nonsteroidal anti-inflammatory drug; PANDAS, pediatric autoimmune neuropsychiatric disorders associated with streptococcal infections; PANS, pediatric acute-onset neuropsychiatric syndrome; SSRIs, selective serotonin reuptake inhibitors.
